# Making large-scale surgical trials possible: collaboration and the role of surgical trainees

**DOI:** 10.1186/s13063-021-05536-7

**Published:** 2021-08-26

**Authors:** Marcus Jepson, Michelle Lazaroo, Samir Pathak, Natalie Blencowe, Jane Collingwood, Madeleine Clout, Giles Toogood, Jane Blazeby, on behalf of the Sunflower Study Executive Group

**Affiliations:** 1 QuinteT Research Group, Population Health Sciences, Bristol Medical School, Canynge Hall, 39 Whatley Road, Bristol, BS8 2PS UK; 2 Clinical Trials and Evaluation Unit, University of Bristol Faculty of Medical and Veterinary Sciences, Bristol, UK; 3 Bristol Centre for Surgical Research, Population Health Sciences, Bristol Medical School, Canynge Hall, 39 Whatley Road, Bristol, BS8 2PS UK; 4 University Hospitals Bristol and Weston NHS Foundation Trust, Trust Headquarters, Marlborough St, Bristol, BS1 3NU UK; 5 Department of Hepatobiliary and Transplantation Surgery, St James’s University Hospital, Leeds, LS9 7TF UK; 6 NIHR Biomedical Research Centre Bristol, University Hospitals Bristol and Weston NHS Foundation Trust, University of Bristol, Oakfield House, Oakfield Grove, Bristol, BS8 2BN UK

**Keywords:** Qualitative research, Optimising RCT recruitment, Trainees, Trial conduct and management, Associate PI scheme

## Abstract

**Background:**

Recruitment to surgical randomised controlled trials (RCTs) can be challenging. The Sunflower study is a large-scale multi-centre RCT that seeks to establish the clinical and cost effectiveness of pre-operative imaging versus expectant management in patients with symptomatic gallstones undergoing laparoscopic cholecystectomy at low or moderate risk of common bile duct stones. Trials such as Sunflower, with a large recruitment target, rely on teamworking. Recruitment can be optimised by embedding a QuinteT Recruitment Intervention (QRI). Additionally, engaging surgical trainees can contribute to successful recruitment, and the NIHR Associate Principal Investigator (API) scheme provides a framework to acknowledge their contributions.

**Methods:**

This was a mixed-methods study that formed a component part of an embedded QRI for the Sunflower RCT. The aim of this study was to understand factors that supported and hindered the participation of surgical trainees in a large-scale RCT and their participation in the API scheme. It comprised semi-structured telephone interviews with consultant surgeons and surgical trainees involved in screening and recruitment of patients, and descriptive analysis of screening and recruitment data. Interviews were analysed thematically to explore the perspectives of—and roles undertaken by—surgical trainees.

**Results:**

Interviews were undertaken with 34 clinicians (17 consultant surgeons, 17 surgical trainees) from 22 UK hospital trusts. Surgical trainees contributed to patient screening, approaches and randomisation, with a major contribution to the randomisation of patients from acute admissions. They were often encouraged to participate in the study by their centre principal investigator, and career development was a typical motivating factor for their participation in the study. The study was registered with the API scheme, and a majority of the trainees interviewed (*n* = 14) were participating in the scheme.

**Conclusion:**

Surgical trainees can contribute substantial activity to a large-scale multi-centre RCT. Benefits of trainee engagement were identified for trainees themselves, for local sites and for the study as a whole. The API scheme provided a formal framework to acknowledge engagement. Ensuring that training and support for trainees are provided by the trial team is key to optimise success for all stakeholders.

## Background

Clinical decision-making is ideally based on the best available evidence [1] from well-conducted and designed randomised controlled trials (RCTs). For patients with symptomatic gallbladder disease being offered laparoscopic cholecystectomy (LC), there is currently a lack of evidence for how to optimally assess the risks of common bile duct (CBD) stones before surgery. Although assessment typically uses a combination of liver function tests (LFTs) and imaging (ultrasound scan (USS) plus or minus magnetic resonance cholangiopancreatography (MRCP)), the clinical benefit and cost effectiveness of undertaking pre-operative imaging for all patients to explore for the presence of CBD stones is unclear, although for patients classified as high risk of stones imaging is generally recommended [2, 3]. This is important because in England alone some 70,000 LCs are undertaken. The Sunflower study [HTA project reference: 16/142/04] [4] is a UK-based multi-centre, pragmatic, unblinded RCT which aims to establish the clinical and cost effectiveness of pre-operative imaging with MRCP versus expectant management (i.e. no pre-operative imaging) in patients with symptomatic gallstones undergoing LC at low or moderate risk of CBD stones.

The Sunflower RCT has an ambitious recruitment target: 13,680 patients randomised to the study in 4 years, from at least 50 UK NHS hospital trusts. To increase the generalisability of the study findings, the sample will include patients who are scheduled for LC as either an elective or urgent procedure. For elective LC patients, it is likely that they will be approached at consultant-led outpatient clinics, with support from trainees and research nurse teams. Urgent admissions may be admitted at any time of the day, including at times when the routine research support infrastructure may not be available. Thus, at the point of trial design, the Sunflower investigators designed the study to include a QRI and administrative support to actively engage surgical trainees using the API scheme. There was a dual purpose: to optimise patient recruitment in the acute setting and to invest in trainees’ research experience to develop capacity. We hoped to create a future group of surgeons who understand and can participate in trials so that they deliver evidence-based practice. In the UK, the National Trainee Research Collaboratives (NTRC) have previously led multi-centre surgical research studies including cohort studies [5–7] as well as RCTs [8–10]. These studies demonstrate the motivation of trainees to take part in research alongside their clinical training [11], in particular where they play a lead role in the study design and implementation. In RCTs with an emergency context, trainees may be more readily available to consent patients, although may benefit from support to do so [12]. Less well reported are studies where trainees have played a parallel role alongside consultant surgeons. Initiatives to encourage trainees to become research active clinicians include the National Institute for Health Research (NIHR) Associate PI (API) scheme. The API scheme was introduced in April 2019, with aims to develop junior doctors, nurses or allied health professionals to be Principal Investigators (PIs) of the future by encouraging and recognising their engagement in NIHR portfolio research [13]. Little is known about how this scheme is working in the context of open trials. The Sunflower study is registered to the API scheme.

The overall aim of this study was to understand factors that supported and hindered the participation of surgical trainees in a large-scale RCT and their participation in the API scheme. We sought to do this by exploring the specific roles undertaken by surgical trainees in the Sunflower study. Additionally, we drew on interviews with consultant surgeons, who, in the context of Sunflower, provide a mentoring role to trainees, to understand what they consider to be the contributions of trainees. We also examined screening and recruitment reports from the study database.

## Methods

### Design

The Sunflower study is a randomised trial that seeks to explore the pre-operative management of patients with symptomatic gallstones. Patients are randomised either to the intervention: a pre-operative magnetic resonance cholangio-pancreaticogram (MRCP) followed by laparoscopic cholecystectomy (LC) surgery or to expectant management (EM) (patients are listed for LC without pre-operative MRCP). Patients are randomised to MRCP or EM in a 1:2 ratio.

The primary outcome of the Sunflower study is any hospital admission within 18 months of randomisation for a complication of gallstones. The study’s first centre opened to recruitment in January 2019. Recruitment paused on 20 March 2020 because of the COVID-19 pandemic. At the time of closing, 48 UK centres were open to recruitment and 1990 patients had been randomised. The pause ended in August 2020, and by 4 January 2021, 2229 patients had been randomised. Recruitment at each centre was led by a site PI (typically a consultant surgeon), with support from surgical trainees, radiologists and research nurses. The study included an embedded QuinteT Recruitment Intervention (QRI). The QRI is a well-established intervention [14] that aims to support challenging RCTs to optimise recruitment and informed consent through a combination of methods, including interviews with RCT recruiting staff. We conducted this exploratory qualitative study, as part of the Sunflower study QRI; additionally, a 50% FTE study trainee administrator was employed to support the trainee involvement and engagement.

### Sampling strategy

The population for the qualitative element of this study was surgical trainees and consultant surgeons registered on the delegation log for the Sunflower study either as site lead trainee or as site PI. Respondents were approached for an interview once their centre had been open to recruitment for 3 months, to give them time to become familiar with the implementation of the study at their centre. The initial approach for the interview was made by the senior study administrator by email, and in cases where there was no response, with at least one further follow-up email. Interviewees were sent a study information leaflet and completed a consent form prior to their interview, or immediately after, having given verbal consent at the start of the recording. Interviews proceeded up to the point when the study was suspended to recruitment in response to the COVID-19 pandemic in March 2020, then continued as sites re-opened in August 2020.

### Data collection

All interviews were undertaken by a male, non-clinically trained, experienced qualitative researcher (MJ). A semi-structured interview topic guide was developed on the basis of the researcher’s experience on other RCTs [15, 16] and in discussion with two clinically trained co-authors (NB and SP). The topic guide included open questions about each respondent’s experience of RCTs and research generally and their specific experience on the Sunflower study. The trainee topic guide additionally included questions about their experiences and opinions on the API scheme. Although the topic guide provided a framework on which to base the interviews, it was not followed rigidly, and should the discussion evolve, or take different directions, this was permitted. Hence, whilst the topic guide was piloted with one interviewee, it was not amended over the course of the study.

All interviews were conducted over the telephone, at a time suited to the respondent. All were audio recorded using encrypted digital recorders and then transcribed verbatim with personal identifiers removed. Screening and recruitment activity, showing admission type (elective or acute), and job role of the person undertaking the assessment of patient eligibility, approach and consent-seeking (consultant, trainee or research nurse) were routinely recorded by study sites and collated centrally on the study database.

### Data analysis

Qualitative data analysis began after the first five interviews were completed and was repeated during the study. Transcripts were checked against recordings for accuracy by MJ and then uploaded to Nvivo 12 [17] for coding. They were not shared with interviewees. Our analytic approach was inductive. Transcripts were analysed using constant comparison methods, with new data compared with earlier examples. Initially, transcripts were coded by MJ against the sections of the interview topic guides. This coding framework was developed iteratively with further themes and codes added during analysis to ensure details not captured in the original frame were not missed. Two additional interviews undertaken after the study re-opened following the pandemic-enforced shutdown did not generate any new codes or data, thus indicating that a notional data saturation had been achieved [18]. MJ wrote descriptive definitions of the themes. These were shared, discussed and agreed with other authors (NB, SP, JC, MC) with expertise in the clinical (as consultant and surgical trainee) and trial management aspects of the study question. Numbers and percentages of patients consented by discipline and according to admission type and screening log activity by discipline were extracted from the study database by ML on 4 January 2021.

## Results

### Participant demographics

Interview invitations were sent to 29 surgical trainees and 30 consultant surgeons at sites that had been open to recruitment for more than 3 months (one site did not have a named lead surgical trainee at the time). Of those approached, 9 declined (4 trainees; 5 consultants) and 16 (8 trainees; 8 consultants) did not respond to the initial request or to two follow-up requests. Invitations were sent once the respective centre had been open to recruitment for at least 3 months, to ensure respondents had the experience of recruiting patients. A total of 34 interviews from 22 recruiting centres were undertaken with 17 surgical trainees (6 female; 11 male) and 17 consultant surgeons (2 female; 15 male) between January 2019 and August 2020. The average interview duration was 25 min (range 15–43 min). Consultant surgeons had been qualified for between 16 and 31 years (mean 22 years). Surgical trainees had been qualified for between 7 and 16 years (mean 11 years).

Four key areas of analytic interest were identified in the data: how trainees became involved in the Sunflower study, trainees’ experiences of engagement with the API scheme, descriptions of the trainees’ roles on the Sunflower study and the benefits trainees derived from study participation.

### How trainees became involved in the Sunflower study

Respondents described how they came to be involved in the Sunflower study. It was rare for trainees to initiate their involvement, and typically, the initiative to take part came about as a result of being put forward or encouraged by the consultant PI at their site. Most commonly, this was driven by the PI recognising a need for support with study recruitment:

[PI name] sent round an email to everyone saying we’re doing this, does anyone want to get involved to help with recruitment? [Trainee 9]

In most cases, trainees interviewed would only expect to be in their current hospital for a maximum of 12 months, although there were circumstances where trainees had a longer rotation. In these cases, this trainee had been asked to take a lead role in the study because of their longer tenure, such as Trainee 5, who expected to remain at the same site for a full year:

[PI name] … asked me if I’d be interested, because I’m going to be there for a while, it’s good for kind of consistency [Trainee 5]

The stated motivating factors for encouraging trainees to take part in Sunflower sometimes went beyond simply supporting study recruitment. In some cases, the site PIs demonstrated a strong belief in a culture of research and had provided encouragement to their trainees to participate as part of their career development. For example, this PI described how they were trying to make a cultural change in their Trust:

I’m a massive advocate for trainees being involved in research and certainly, you know, basically every time I’m operating with a new trainee I talk to them – have you got GCP, you want to get involved in these studies. Obviously, I don’t try to completely force people into – just trying to shift the culture. I still look at the lists – ST3 and ST1 regional teaching things about options of getting involved in research including things like Sunflower. To try and promote getting people involved. [Consultant 15]

### Engagement with the API scheme

In order to complete the API scheme, participants must have been a member of the site research team for a minimum of 6 months. They must complete an online learning course and record evidence of their participation in the RCT on the API scheme status checklist (see the Appendix) against core (mandatory) and additional (non-mandatory) activities, which is checked and authorised by the local PI and the Trial manager. Core activities are categorised as team activities (through being named on the study delegation log; disseminating the study at the local site; meeting regularly with the PI and local study team; supporting the PI and attending local research meetings), study management/compliance activities (checking screening logs) and patient-related activities (being involved in consenting patients to the RCT). Additional activities include interacting with the clinical trial unit, standing in for the PI at study meetings, collaborating with other APIs and involvement in PPI activities.

At the time this paper was written, 34 trainees, from 30 Sunflower centres, had engaged with the API scheme in some form. In some cases, this simply involved them approaching the trainee administrator to ask for information about the scheme in relation to the Sunflower study:

I’ve signed up for it officially but I haven’t, I haven’t been through the paperwork with ((PI name)). That’s something I need to do in due course. And the one thing I’ve struggled with, and I think that’s my fault more than anything [Trainee 6]

Contrastingly, others talked about completing some, or all of the relevant documentation to achieve certified API status.

I’ve done all the things properly - I haven’t actually put my - I didn’t put my NIHR paperwork in …. So broadly speaking yes, I’ve been, sort of, delegated the roles but I haven’t got the official - I haven’t done the official NIHR paperwork. [Trainee 7]

Motivations for participating in the API scheme were dominated by the perceived career benefit it would bring:

Interviewer: What was your motivation for signing up for the scheme?

Trainee 3: I read up on it and saw that it would be good because you will be recognised, it would be an extra recognition of my work. Nowadays for our CVs it’s quite important to have evidence … that you’ve done something … and you’ve taken part in the study.

so I’m coming to the end of my training and hope to become a consultant soon, …it’s [the API role] something I’m definitely hoping to talk about at the interview. … I think the associate PI scheme has really encouraged me to feel a part of the team [Trainee 6]

In another case, whilst their professional role had not changed, this interviewee appreciated the status associated with having the title of API:

Having the title, I think the role that I play would probably not have been too different without a title. But having a title I think recognises you that you’re doing this job specifically [Trainee 10]

The same respondent also suggested that having a ‘named’ API could benefit the study also, by providing a more available contact point than, for example, a consultant PI:

It’s also useful as a person as a contact to go to…. I think people locally who know that the Trainee PI is out there and probably more approachable and probably more free with my time there than the consultant PI might be [Trainee 10]

In one case, participation for a more experienced trainee had been driven by an interest in the research generally and the research question in the study specifically:

does (the API Scheme) affect me going forward in terms of my CV? I don’t think it really makes a huge amount of difference. I do think being involved in these studies and being actively involved over a period of time makes a difference. And I actually believe in the studies as, sort of, corny as that sounds. I actually quite like research and I like this study, I think it’s a really good study. So I wasn’t hugely drawn in by the title or the role but was happy to take it on to try and drive the study. [Trainee 7]

Of those interviewed, only one had made a conscious decision to not join the API scheme; that trainee did not see how they would benefit from it:

I don’t see what it would add to my (career)… my role wouldn’t change that much considering that I’m the trainee lead here even if I did the associate PI scheme. I don’t understand how because ((Consultant)) would still be leading it. [Trainee 1]

### Trainees’ contributions to the study

From the outset of the study, it was expected that surgical trainees’ main contribution to the study would be through the recruitment of patients in emergency contexts. This expectation was confirmed by PIs and trainees alike:

So, when [trainee name] is on call he can look after the emergency admissions, and pick up patients through that [Consultant 1]

… (the trainees) are mainly picking up emergency ones, so they’re helping on that front. [Consultant 16]

Essentially [my role is] … particularly finding acute patients whenever I was on call … highlighting that these patients for example were suitable for the study, highlighting that to the consultant… And then recruiting for the study [Trainee 3]

However, surgical trainees also provided support to outpatient elective clinics, either shadowing consultants or leading the study in those contexts:

[Trainee] works with the other two consultants as well and does their clinics, so I’m hoping that [trainee] will start recruiting their patients and that might feed upwards to the other consultants [Consultant 6]

Screening log data showed the proportion of patients consented to the study by surgical trainees was proportionally higher in acute admissions (Table 1).

**Table 1:** Screening log consent by discipline and admission type (data extracted 4 January 2021)

	Elective (of 1665 patients)		Acute (of 504 patients)	
	*N*	%	*n*	%
Consultant	291	17%	89	18%
Trainee	185	11%	176	35%
Res nurse/practitioner	1189	71%	239	47%

Admission type data was not available for all patients, hence a discrepancy of 60 patients between totals consented in Tables 1 and 2

In addition to consenting patients to be randomised to the study, trainee roles also included screening hospital lists, assessing patients against the study inclusion criteria and completing study paperwork—for example, screening logs and entering data onto the study database. One trainee described his involvement in the study and his liaison with others:

I’m going through the inclusion criteria in clinic, … [consultant name] will actually go through his clinic list in advance too and help identify potential recruits, then we’ve got a very good research nurse who is very heavily involved, and sometimes she’ll come down to the clinic to give a hand as well… And once we’ve identified [an eligible patient] we’ll usually broach the subject and just explain a bit about the role of MRCP and then explain that it’s uncertain whether it adds any clinical benefit for patients who’ve got normal or normal-ish liver function tests and whether they have to take part in a study to identify whether it is of benefit to help themselves and future patients. [Trainee 5]

This collaborative approach was common among the sample. A consultant PI described how another trainee had been working with the team in their trust on the study who wanted to develop their role to also screen lists of patients as well as approaching them in the clinic:

I’ve got (trainee name) who’s our trainee lead for this. And I’ve had another registrar who has actively recruited the patients, and she’d rung me up and said come and show me how to do this because I need a hand, but she’s been very competent as well. So, that’s all been good [Consultant 10]

The study database recorded who had undertaken four stages of activity, which included (i) identifying patients (screened), (ii) providing study information (provided PIL), (iii) having initial discussions (approached), through to (iv) receiving their consent (consented) (Table 2). The highest level of activity entered on the database was by research nurses or practitioners. As can be seen in Table 2, trainee and consultant activity levels were very similar, with trainees screening more patients than consultants (17% of all v 11%). These quantitative data support the experiences described in the qualitative interviews. It should be noted that not all activities were undertaken by the same person. For example, it is plausible that a patient could be screened by a trainee, provided with a PIL by a research nurse, approached by a consultant and consented and randomised by a research nurse.

**Table 2:** Screening log activity by discipline (data extracted 4 January 2021)

	Patients screened (of 4525 entries)		Provided PIL (of 3078 entries)		Approached (of 2864 entries)		Consented (of 2229 entries)	
	*N*	%	*N*	%	*N*	%	*N*	%
Consultant	525	11%	504	16%	483	17%	394	18%
Trainee	758	17%	401	13%	441	15%	375	17%
Res nurse/others	3242	72%	2173	71%	1940	68%	1460	65%

### Benefits that trainees derived from study participation

In addition to the benefits of participating in the API scheme, trainees also reported a range of benefits from being involved in the Sunflower RCT. These were most commonly in relation to their career development. For example, they envisaged that they would be able to document RCT involvement on their CV—including completion of Good Clinical Practice (GCP) training—and this would benefit their career aspirations as ‘research active clinicians’ and potentially to become a centre PI in their own right.

I think it [taking part in Sunflower] certainly would look good on the CV and also gear me up to getting more involved in research in the future, exploring these options of ways to expand my career [Trainee 5]

Consultant surgeons also identified career benefits for their trainees. For example, some stated that RCT participation would contribute to the trainees’ ability to enter the Specialist Register through completion of their Certificate Completion of Training (CCT):

Because basically for trainees it used to be that you had to write three papers to get the CCT, but now you just have to show evidence of collaboration in research and recruiting patients does count. [Consultant 4]

ultimately, it’s about learning and training and saying you’ve recruited to studies … actually being able to say, “I have screened people for an RCT, I’ve consented them, I did the training to do that,” is now considered a much more important skills in terms of your training and the criteria you need to meet to progress within the different training programmes [Consultant 17]

In other instances, particularly for those who intended to pursue a clinical specialism in hepatobiliary or upper GI surgery, trainees were invested in the outcome of the Sunflower study. Thus, by contributing to the study’s recruitment, they saw themselves as helping to inform clinical practice, either in general or specifically in their intended clinical specialty:

I think there’s a lot of inefficiency in the NHS and so this is something that provides clear black and white answers to – sometimes what seems like inefficiency. [Trainee 13]

I mean, if we manage to find out that a significant, or a proportion of the MRCPs that we organise are not useful, you know, that’s a big achievement I think. [Trainee 12]

it’s a clinically incredibly important [research] question and relevant and so that’s what I like about the study. It has potential to really change practice, either way. You know, it could really show, could really show that an MRCP is a really important safety feature of the way we manage these patients. It’s really important to know that [Trainee 7]

## Discussion

This paper has explored how surgical trainees have been integrated into the NIHR Sunflower study and the NIHR API scheme. It aims to understand factors that supported the participation of surgical trainees in a large-scale RCT and their participation in the API scheme. We developed several themes from our qualitative analysis of interviews with 34 clinicians (consultant and trainee surgeons) which were that trainees typically became involved in the Sunflower study as a consequence of being encouraged to do so by the consultant lead at their local site or through prompting from the study trainee administrator. Many of the trainees interviewed (14 of 17) had engaged with the NIHR API scheme as part of their role on the Sunflower study, citing the potential benefit to their career development. Trainees had been involved in all phases of recruitment to the study: screening clinic lists, assessing for eligibility against the study inclusion and exclusion criteria, approaching patients and seeking their consent to be randomised. Trainee’s contribution to the study had taken place in both elective and acute settings. From the beginning of the study to January 2021, 35% of all acute patients (*n* = 185) and 11% of elective patients (*n* = 175) were consented to the study by a trainee. We may deduce from these figures that recruitment to the study would have been significantly lower without these substantial contributions, in particular in acute settings. As well as gaining career benefits from participation in the Sunflower study, those with an HPB or upper GI specialty were invested in contributing to a study with the potential to change clinical practice in their discipline. Embedding the API scheme into a large-scale RCT to optimise recruitment and invest in future research capacity is therefore recommended as a useful contribution.

The success of multi-site RCTs relies on the engagement of local research teams [19] and, in common with our findings, the role of a consultant champion has been considered valuable to support trainees’ engagement in RCTs [20]. The role of consultant-as-mentor can have a positive benefit on trainees’ career development, particularly where the consultant shows an ‘investment’ in the trainee [21]. In Sunflower, a number of consultants demonstrated that they were considering their trainee colleague’s future plans, pinpointing the types of research skills needed to aid their progression. In Sunflower, additional resources were allocated to enable trainee engagement. Prior to each site initiation, the study trainee administrator emailed the site PI to encourage them to identify a trainee who may act as lead for their centre. Additionally, at site initiation meetings, the potential benefit to the RCT of having trainee collaboration was outlined, in particular their recognition on study publications. Whilst we acknowledge as a limitation that we did not explore in detail trainees’ views on the role of the trainee administrator, we suggest that the designated central administrative support for trainee collaborations, in addition to core trial management, contributed to the successful engagement of trainees in the study. The administrator tracked trainees as they rotated between sites, providing advantages for the study: trainees ensured continuity by taking their experience to their new site, and for the trainee: their involvement in the study could last longer and thus provide further opportunities to rejoin the API scheme and acquire RCT-related skills.

The Sunflower RCT had more trainees registered with the API scheme than any other study in the UK at the time of this study (although subsequently, co-authors NB and JMB have introduced the API Scheme into the COVID RECOVERY trial with a higher number of APIs involved). The majority of those interviewed were participating in the scheme, although some had yet to complete the requisite tasks or paperwork to achieve completion of the scheme. Career development was the most commonly cited reason trainees gave for engaging with the API scheme. Completing the scheme’s paperwork was seen by some as being burdensome. In a context where surgical trainees report finding work-life balance challenging [22], this may be off putting to some. The study’s trainee administrative support helped to reduce the burden on trainees in relation to the API scheme. Providing administrative support is recognised as a key component of good trial conduct generally [23], we reflect that the additional and specific administrative support in place to co-ordinate the API scheme, and the involvement of trainees more widely, was something that may help other, large-scale studies, to successfully engage with surgical trainees and thus enhance recruitment potential.

Trainees reported that they had undertaken a range of activities on the Sunflower RCT. This included screening clinic lists, assessing patients’ eligibility against the study inclusion criteria, discussing the study with them and logging these activities on the study database. In the RCT set-up phase, case report forms (CRFs) were designed with reference to the SEAR framework [24]. This recommends documenting different activities (numbers of patients screened, eligible, approached and randomised) as a means of closely monitoring RCT activity, in order to identify any recruitment obstacles. Research nurses had apparently undertaken the majority of each logged study activity (screening, providing PILs, approaching and consenting patients). Anecdotal evidence from informal discussions with a sub-set of research nurses suggested that this may, in part, have been a result of their role transferring case report forms (CRFs) onto the study database, a role commonly undertaken by clinical research nurses [25]. What the data clearly evidence is that the logged contribution of trainees and consultants in each of those four areas was almost identical. When we compared activity for elective and acute patients, we observed a marked difference in activity levels. Trainee surgeons consented a substantially higher proportion of the 504 acute patients, than those consented by consultant surgeons (35% v 18%). Trainees have contributed to cohort studies, involving acute and elective patients [26]. This paper demonstrates their contributions to recruitment in an RCT in a similar context.

Analysis of the qualitative data illustrates the reciprocal benefits derived by the trainees on this RCT. Being part of a large-scale national RCT would reflect well for the participants’ career development. A modified Delphi study [27] identified two core and four additional requirements of academic achievement for surgical trainees to gain their certificate of completion of training (CCT). Active trainee participation in the Sunflower study would result in trainees meeting at least one core requirement (undertaking GCP training) and one additional requirement (recruiting patients to a multi-centre study). The application of the SEAR framework [24] (described above) allowed a means of quantifying recruitment and related activities by trainees and other staff. Recording activity levels such as this can assist with enabling a fair collaborative authorship model, a challenge in large-scale multi-centre RCTs [28]. Being part of an accredited scheme—in this case, the NIHR API award—was seen to offer a more formal, demonstrable benefit than simply being involved in the conduct of the RCT without that recognition. The benefits of developing a cohort of research-trained and experienced surgical trainees fit well with a wider investment in evidence-based surgery [29] and will enhance the potential of a ‘collaborative’ research-focussed surgical workforce [30]. Recruitment to RCTs can be a complex and challenging process [31] and a teamworking approach, such as that described here, can help to mitigate some of these challenges [32].

### Strengths and limitations

As far as we are aware, this is the first qualitative study that has described the involvement and experiences of surgical trainees involved in recruitment to a national RCT. The respondents were experts by virtue of being either surgical trainees or consultants involved in supporting trainees. All participants had recruitment experience on the Sunflower RCT. We acknowledge that the respondents were all relatively engaged with the study, and the majority of the trainees, with the API scheme. We may speculate that those trainee and consultant surgeons who declined participation in this interview study were less engaged with this programme. Our recommendation that administrative support is in place to encourage trainee involvement and engagement inevitably relies on a study having the necessary infrastructure and funding in place to do so. Trainees interviewed did not report any substantial barriers to participating in the RCT, or in relation to the API scheme, nor did they indicate any reluctance to be involved in the study. We reflect that the sample represented here were those with an interest in RCTs and this is a limitation. Future work may usefully explore reasons why trainees do *not* participate in RCTs or the API scheme.

## Conclusions

This study makes a useful contribution to our understanding of the motivations that encourage surgical trainees to take part in a national RCT, the ways in which they become involved in an RCT and the mechanisms needed to support them in their role. We conclude that trainees can, and do, play a key role in supporting the conduct of multi-centre large-scale RCTs and this role provides benefits on a number of levels. In the Sunflower study, at a local level, trainees provided infrastructure to assist consultants and research nurses in screening, identifying and consenting patients. This in turn benefitted the RCT as a whole; with more personnel engaged in study activity, recruitment numbers and rates were higher. Trainees derived career development benefits from involvement in a large-scale multi-site RCT, with the potential to contribute to changing clinical practice. The NIHR API scheme provides trainees with a formalised, nationally recognised framework to gain research skills and demonstrate their engagement with an RCT. As the API scheme is a relatively new initiative, further research may be useful to monitor how it is integrated into practice. At present, the scheme is being extended from surgical trials to all specialities. The NIHR provides central support and information is available on the NIHR website, but there are important implications for the trial units that host trials including API schemes must consider. Future work is needed in particular to explore any barriers to implementation of the API scheme because of increased administrative work for trial units.

## Data Availability

The datasets that we have acquired will not be available as our ethical approval does not permit the sharing of qualitative datasets.

## Appendix

**Table 3:** Consultant gender and specialty

Consultant	Gender	Specialty	Years qualified
1	M	General/upper GI	21
2	M	General/emergency	19
3	M	General/colorectal	31
4	M	General/upper GI	19
5	M	General/upper GI	18
6	M	Upper GI	15
7	M	General/upper GI	31
8	F	Upper GI	19
9	M	Upper GI	20
10	M	Upper GI	29
11	M	General/upper GI	18
12	M	Upper GI	19
13	M	Upper GI	24
14	M	Upper GI	20
15	F	Upper GI	16
16	M	General/upper GI	30
17	M	HPB	23

**Table 4:** Trainee gender, grade and specialty

Trainee	Gender	Role/specialty	Grade	Years qualified
1	M	Upper GI/bariatrics	ST5	7
2	F	General surgery	ST4	11
3	M	General surgery	ST3	10
4	F	Colorectal/all upper GI	Staff grade	8
5	M	General surgery	Trust grade	7
6	M	Upper GI	ST6	10
7	M	Upper GI	ST7	10
8	M	General surgery	ST6	9
9	F	Upper GI	Staff grade	8
10	M	HPB	ST5	9
11	M	Upper GI	ST7	15
12	M	Upper GI	Post-CCT	14
13	F	General surgery	CP2	16
14	M	HPB	ST8	14
15	M	CRS	ST4	9
16	F	General	ST4	9
17	F	HPB	ST8	12
