# Correction to: Making large-scale surgical trials possible: collaboration and the role of surgical trainees

**DOI:** 10.1186/s13063-021-05609-7

**Published:** 2021-09-13

**Authors:** Marcus Jepson, Michelle Lazaroo, Samir Pathak, Natalie Blencowe, Jane Collingwood, Madeleine Clout, Giles Toogood, Jane Blazeby, on behalf of the Sunflower Study Executive Group

**Affiliations:** 1 QuinteT Research Group, Population Health Sciences, Bristol Medical School, Canynge Hall, 39 Whatley Road, Bristol, BS8 2PS UK; 2 Clinical Trials and Evaluation Unit, University of Bristol Faculty of Medical and Veterinary Sciences, Bristol, UK; 3 Bristol Centre for Surgical Research, Population Health Sciences, Bristol Medical School, Canynge Hall, 39 Whatley Road, Bristol, BS8 2PS UK; 4 University Hospitals Bristol and Weston NHS Foundation Trust, Trust Headquarters, Marlborough St, Bristol, BS1 3NU UK; 5 Department of Hepatobiliary and Transplantation Surgery, St James’s University Hospital, Leeds, LS9 7TF UK; 6 NIHR Biomedical Research Centre Bristol, University Hospitals Bristol and Weston NHS Foundation Trust, University of Bristol, Oakfield House, Oakfield Grove, Bristol, BS8 2BN UK

**Correction to: Trials 22, 567 (2021)**

**https://doi.org/10.1186/s13063-021-05536-7**

Following the publication of the original article [1], we were notified of a missing statement in the Funding section.

- Originally published Funding section read:

The study is also supported by the Royal College of Surgeons Surgical Trials Centre in Bristol. NB and JB are supported by the NIHR Bristol and Weston Biomedical Research Centre. (etc.....)

- The Funding section should actually read:

**This work is supported by the National Institute for Health Research (NIHR) Health Technology Assessment Programme (Grant Ref: 16/142/04).** The study is also supported by the Royal College of Surgeons Surgical Trials Centre in Bristol. (Etc...)

The original article has been corrected.

## References

[1] Jepson (2021). Making large-scale surgical trials possible: collaboration and the role of surgical trainees. Trials.

